# The role of *NFKB1* and *NFKBIA* in immunoglobulin A vasculitis

**DOI:** 10.3389/fimmu.2025.1692908

**Published:** 2025-10-24

**Authors:** Joao Carlos Batista-Liz, María Sebastián Mora-Gil, Mónica Renuncio García, María Teresa Leonardo, Ana Peñalba, Ligia Gabrie, Rafael Gálvez Sánchez, Luis Martín-Penagos, Javier Narvaez, Belén Sevilla-Pérez, Raquel Ríos Fernández, José Luis Callejas-Rubio, Luis Caminal-Montero, Paz Collado, José Javier Pérez Venegas, María José Rodríguez Valls, Diego De Árgila, Patricia Quiroga Colina, Esther Francisca Vicente Rabaneda, Esteban Rubio, Manuel León Luque, Juan María Blanco-Madrigal, Eva Galíndez-Agirregoikoa, Javier Gonzalo Ocejo-Vinyals, Ricardo Blanco, Verónica Pulito-Cueto, Raquel López-Mejías

**Affiliations:** ^1^ Immunopathology Group, Instituto de Investigación Marqués de Valdecilla (IDIVAL), Santander, Spain; ^2^ Immunology Unit, Complejo Asistencial Universitario de León, León, Spain; ^3^ Division of Paediatrics, Hospital Universitario Marqués de Valdecilla, Santander, Spain; ^4^ Division of Rheumatology, Hospital Universitario Marqués de Valdecilla, Santander, Spain; ^5^ Division of Nephrology, Hospital Universitario Marqués de Valdecilla, Santander, Spain; ^6^ Division of Rheumatology, Hospital Universitario de Bellvitge, Barcelona, Spain; ^7^ Division of Paediatrics, Hospital Universitario Clínico San Cecilio, Granada, Spain; ^8^ Systemic Autoimmune Disease Unit, Hospital Universitario Clínico San Cecilio, Instituto de Investigación Biosanitaria Ibs.GRANADA, Granada, Spain; ^9^ Internal Medicine Department, Hospital Universitario Central de Asturias, Instituto de Investigación Sanitaria del Principado de Asturias (ISPA), Oviedo, Spain; ^10^ Division of Rheumatology, Hospital Universitario Severo Ochoa, Madrid, Spain; ^11^ Division of Rheumatology, Hospital General de Jerez, Jerez de la Frontera, Spain; ^12^ Division of Dermatology, Hospital Universitario de La Princesa, Madrid, Spain; ^13^ Division of Rheumatology, Hospital Universitario de La Princesa, Instituto de Investigaciones Sanitarias (IIS)-Princesa, Universidad Autónoma de Madrid, Madrid, Spain; ^14^ Division of Rheumatology, Hospital Universitario Virgen del Rocío, Sevilla, Spain; ^15^ Division of Rheumatology, Hospital Universitario de Basurto, Bilbao, Spain; ^16^ Department of Immunology, Hospital Universitario Marqués de Valdecilla, Santander, Spain; ^17^ Infectious Diseases and Clinical Microbiology Group, Instituto de Investigación Marqués de Valdecilla (IDIVAL), Santander, Spain

**Keywords:** biomarkers, immunoglobulin A vasculitis (IgAV), NF-kappa B (NF-kB), *NFKB1*, *NFKBIA*

## Abstract

**Introduction:**

Immunoglobulin A vasculitis (IgAV) is an inflammatory disease mediated by B cells. Nuclear factor kappa B (NF-κB) is essential for B-cell development and maturation and plays a key role in autoimmunity and inflammation. In particular, the NF-κB canonical activation pathway genes *NFKB1* (encoding NF-κB1) and *NFKBIA* (encoding NF-κB inhibitor alpha) have been identified as risk *loci* for several immune-mediated diseases, but their role in IgAV remains unclear. This study aimed to determine whether *NFKB1* and *NFKBIA* represent novel genetic risk factors for IgAV pathogenesis.

**Methods:**

The *NFKB1* promoter variant −94 ins/del ATTG (rs28362491), six tag *NFKB1* polymorphisms (rs77830930, rs1598856, rs7340881, rs4648055, rs4648090, and rs230547), and seven tag *NFKBIA* variants (rs3138055, rs696, rs1022714, rs2233419, rs2233415, rs1050851, and rs1957106) were genotyped in 343 Caucasian IgAV patients and 764 healthy, ethnically matched controls using TaqMan probes. Patients were stratified according to age at disease onset and the presence or absence of renal, articular, and gastrointestinal manifestations. Genotype, allele, and haplotype frequencies were compared between patients and controls, as well as across clinical subgroups.

**Results:**

No statistically significant differences were found in genotype or allele frequencies of *NFKB1* or *NFKBIA* between IgAV patients and healthy controls. Likewise, haplotype frequencies of both genes were similar across groups. No associations were observed when patients were stratified by clinical features, including renal involvement, age at onset, or articular/gastrointestinal symptoms.

**Conclusion:**

Our findings do not support a major role for the *NFKB1* or *NFKBIA* variants studied in IgAV susceptibility or severity. These results suggest that if NF-κB signaling contributes to IgAV pathogenesis, it likely involves other biological mechanisms.

## Graphical Abstract

Graphical summary of the study design, genotyping analyses, and main findings on NFKB1 and NFKBIA polymorphisms in IgA vasculitis. Some icons in this figure were adapted from Flaticon (www.flaticon.com).

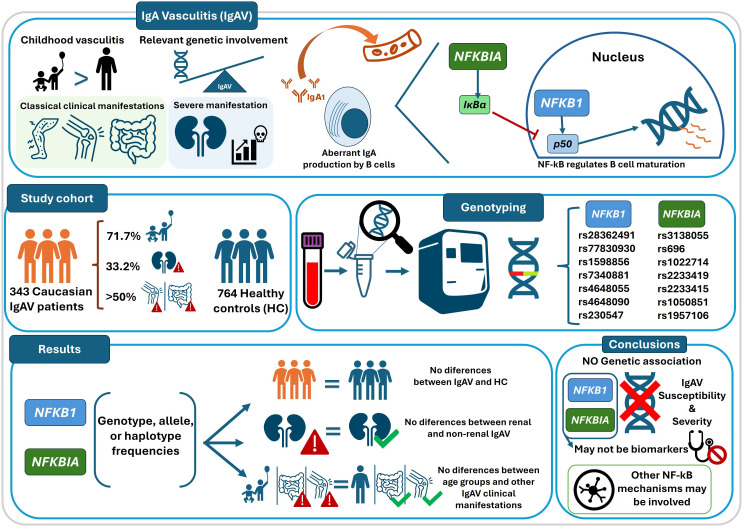

## Introduction

Immunoglobulin A vasculitis (IgAV) is an inflammatory disease characterized by the deposition of immune complexes of aberrantly glycosylated IgA1 in the walls of small blood vessels, leading to inflammatory responses and tissue injury (1–3). IgAV occurs more frequently in children and is the most common systemic vasculitis in the pediatric population (4, 5), although adults can also be affected (6, 7). The most common clinical manifestations of IgAV are the classic triad of palpable purpura, arthralgia/arthritis, and gastrointestinal (GI) symptoms (8–10). However, the most life-threatening complication is the development of renal manifestations (2, 3, 11). In this regard, adult patients have poorer outcomes than children, as they are more likely to experience chronic renal damage and progress to end-stage renal disease (3, 6, 12). Moreover, the severity of renal involvement does not always correlate with the initial presentation of the disease (2, 10, 13). Accurate diagnosis of IgAV and confirmation of nephritis require skin and renal biopsy, which are highly invasive procedures. Consequently, diagnosis is often delayed, and early detection of nephritis is difficult, leading to poorer patient outcomes (14). Identifying reliable disease biomarkers could therefore provide a minimally invasive complementary tool to support earlier and more accurate diagnosis, as well as to enable the detection and treatment of severe nephritis cases in the initial stages of renal injury.

Although the pathogenesis of IgAV remains largely unknown, it has been described as a B cell–mediated disease (2). Multiple studies, including our previous work, have highlighted the importance of genetic factors in its pathogenesis (3, 9, 11). Notably, our earlier studies indicated that polymorphisms in B cell activation and signaling genes—such as *BAFF*, *APRIL*, *BAFFR*, *CD40*, *BLK*, and *BANK1*—do not appear to play a significant role in IgAV development (15, 16). However, given the established involvement of B cells in the disease, it is reasonable to hypothesize that other genes regulating B cell function may contribute to IgAV pathogenesis.

In this context, nuclear factor kappa B (NF-κB) is a known regulator of multiple physiological processes and plays a central role in the activation, response, and survival of mature B cells, as well as in the regulation of class switching that leads to IgA production (17–22). Specifically, NF-κB1 (p105), encoded by the *NFKB1* gene located on chromosome 4q24, is one of the principal members of the NF-κB family of proteins (23). Following proteolytic processing, NF-κB1 generates p50, the central component of the canonical NF-κB activation pathway (18). The activity of p50 is regulated in the cytoplasm by NF-κB inhibitor alpha (IκBα), encoded by the *NFKBIA* gene located on chromosome 14q13 (24). NF-κB has been implicated in the pathogenesis of several pediatric autoimmune diseases. In IgAV, tumor necrosis factor–like weak inducer of apoptosis activates NF-κB through IκBα phosphorylation, while studies in juvenile systemic lupus erythematosus and juvenile systemic sclerosis have reported elevated NF-κB levels and reduced peroxisome proliferator-activated receptor gamma expression, further supporting the role of NF-κB–mediated proinflammatory signaling in childhood-onset autoimmunity (25, 26). Interestingly, genetic variants in both *NFKB1* and *NFKBIA* have been proposed as risk *loci* for multiple immune-mediated diseases (24, 27–31) and, in particular, for other systemic vasculitides such as Behçet’s disease (32). However, the role of these genes in the pathogenesis of IgAV remains to be elucidated.

Considering these precedents, the present study aims to elucidate, for the first time, whether polymorphisms in *NFKB1* and *NFBKIA* are associated with susceptibility to IgAV or with specific clinical and demographic manifestations of the disease, and to explore their potential value as candidate biomarkers in a large, well-characterized cohort of IgAV patients and controls.

## Methods

### Study population

This study, conducted between 2012 and 2024, included a large multicenter cohort of 343 unrelated Spanish patients diagnosed with IgAV who met the criteria established by Michel *et al.* (33) and the American College of Rheumatology classification criteria for IgAV (34). Patients were recruited from nine Spanish hospitals: Hospital Universitario Marqués de Valdecilla (Santander), Hospital Universitario Clínico San Cecilio (Granada), Hospital Universitario de Bellvitge (Barcelona), Hospital Universitario Central de Asturias (Oviedo), Hospital Universitario Severo Ochoa (Leganés), Hospital Universitario de La Princesa (Madrid), Hospital Universitario Virgen del Rocío (Seville), Hospital General de Jerez (Jerez de la Frontera), and Hospital Universitario de Basurto (Bilbao). Clinical and demographic data for all participants were collected. The final sample size represents all patients who met the inclusion criteria and were available during the recruitment period across the nine participating centers. Patients were considered to have renal manifestations if they presented hematuria, proteinuria, nephrotic syndrome, or any combination thereof at any time during the disease course and if renal sequelae (persistent renal involvement) were documented at their last follow-up. Articular manifestations were defined by the presence of arthralgia and/or arthritis, and gastrointestinal (GI) manifestations by the occurrence of bowel angina, GI bleeding, or both. For subsequent analyses, IgAV patients were stratified according to age at disease onset (children ≤20 years; adults >20 years) and the presence or absence of renal, articular, and GI manifestations.

In addition, 764 unrelated, ethnically matched individuals without a history of cutaneous vasculitis or other autoimmune diseases were included as healthy controls. These controls were recruited from Hospital Universitario Marqués de Valdecilla (Santander, Spain) and the National DNA Bank Repository (Salamanca, Spain). All patients with IgAV and healthy controls provided written informed consent. The study procedures followed approved guidelines, regulations, and ethical standards in accordance with the principles of the Declaration of Helsinki. The protocol was approved by the Institutional Review Board or Ethics Committee for Clinical Research of Cantabria, Spain.

### Single nucleotide polymorphism selection

For this study, the *NFKB1* −94 ins/del ATTG (rs28362491) single nucleotide polymorphism (SNP), previously associated with other immune-mediated diseases (28, 30), was selected. In addition, comprehensive tagging of *NFKB1* and *NFKBIA* was performed using data from the 1000 Genomes Project (http://www.internationalgenome.org/) and the Haploview v4.2 software, considering an r^2^ threshold set at 0.8 and a minimum minor allele frequency (MAF) of 0.10. As a result, six tag SNPs located in *NFKB1* (rs77830930, rs1598856, rs7340881, rs4648055, rs4648090, and rs230547) and seven tag *NFKBIA* SNPs (rs1957106, rs1050851, rs2233415, rs2233419, rs1022714, rs696, and rs3138055) were selected, covering most of the variability in both genes.

### Genotyping

Genomic deoxyribonucleic acid (DNA) was extracted from peripheral blood samples of all individuals included in this study using the REALPURE “SSS” kit (RBME04, REAL, Durviz S.L., Spain), based on a salt precipitation method involving selective protein precipitation with a high-salt solution and DNA recovery by alcohol precipitation. All individuals were genotyped for the 14 genetic variants mentioned above using TaqMan genotyping probes (C_61632788_30 for rs28362491, C_102965997_10 for rs77830930, C:_3066480_10 for rs1598856, C:_3066465_10 for rs7340881, C:_3066440_10 for rs4648055, C:32331236_20 for rs4648090, C: 3066430_10 for rs230547, C:_2797261_1_ for rs1957106, C:_2797260_1_ for rs1050851, C::170225_20 for rs2233415, C::165701_10 for rs2233419, C:_7581384_10 for rs1022714, C::145669_30 for rs696, and C:27468306_10 for rs3138055) by real-time polymerase chain reaction (qPCR) in a QuantStudio™ 7 Flex qPCR system (Applied Biosystems, Foster City, CA, USA), according to the manufacturer’s recommended conditions. The corresponding context sequence of each Applied Biosystems TaqMan assay used in this study is provided in **Supplementary Table S1**.

Negative controls and duplicate samples were included to validate genotyping accuracy.

The *NFKB1* and *NFKBIA* genotypes were analyzed to verify conformity with Hardy–Weinberg equilibrium (HWE).

### Statistical analysis

An association analysis comparing the *NFKB1* and *NFKBIA* genotype and allele frequencies between IgAV patients and healthy controls, and among IgAV patients stratified according to the presence of renal manifestations, age at disease onset, and the presence or absence of articular and GI manifestations, was performed using nonparametric tests (chi-squared test or Fisher’s exact test when expected values were <5). Analysis of allelic combinations (haplotypes) was conducted for the seven *NFKB1* SNPs and the seven *NFKBIA* SNPs using Haploview v4.2 software. Haplotype frequencies were calculated, and those with a frequency greater than 5% were selected and compared between the groups mentioned above using the chi-squared test. The strength of all associations was estimated using odds ratios (ORs) and 95% confidence intervals (CIs).

The two-tailed p-values obtained from all statistical analyses were corrected for a false discovery rate (FDR) of 0.05 using the Benjamini–Hochberg procedure. FDR-corrected p-values less than 0.05 were considered statistically significant.

All statistical analyses were performed using STATA statistical software 12 (Stata Corp., College Station, TX, USA). Statistical power calculations were performed with QUANTO v1.2.4 under a log-additive model, using our study design (343 cases and 764 controls, α = 0.05, two-sided). Power was estimated at different ORs and minor allele frequencies (MAFs), considering the tagging threshold of MAF = 0.10 as well as more common variants (MAF ≥ 0.20).

## Results

### Patient characteristics

Among the 343 patients with IgAV, 71.7% (246) were children (≤20 years), 33.2% (114) developed renal manifestations, 55.1% (189) presented articular involvement, and 51.0% (175) showed GI complications. **Supplementary Table S2** summarizes the main demographic characteristics and clinical manifestations of the IgAV patients.

### Genotyping results

A genotyping success rate above 98% was achieved for all *NFKB*1 and *NFKBIA* polymorphisms. All polymorphisms in *NFKB1* and *NFKBIA* were in Hardy–Weinberg equilibrium (HWE) in healthy controls (p > 0.05 in all cases).

The genotype and allele frequencies of all polymorphisms studied in *NFKB1* and *NFKBIA* among healthy controls were consistent with those reported in the 1000 Genomes Project (https://www.internationalgenome.org/) for European populations.

For our sample size, statistical power was 66% for OR = 1.4 and 82% for OR = 1.5 when MAF = 0.10. For more common variants (MAF ≥ 0.20), power exceeded 80% to detect OR ≥ 1.4.

### Association analysis of *NFKB1* and *NFKBIA* polymorphisms between IgAV patients and controls

To determine the influence of *NFKB1* and *NFKBIA* on IgAV susceptibility, we compared the genetic frequencies of these genes between IgAV patients and healthy controls.

In this respect, comparison of the genotype and allele frequencies of the seven *NFKB1* and seven *NFKBIA* polymorphisms yielded no statistically significant differences between IgAV patients and healthy individuals (**Tables 1**, **2**).

**Table 1:** Differences in genotype and allele frequencies of *NFKB1* between IgAV patients and healthy controls.

SNP	Genotype, % (n)/allele, % (2n)	IgAV patients	Healthy controls	P	OR [95% CI]
rs28362491	Ins^1^/Ins^1^	41.7 (140)	37.2 (278)	–	Ref.
	Ins^1^/Del^2^	45.8 (154)	47.7 (357)	0.27	0.86 [0.65 - 1.13]
	Del^2^/Del^2^	12.5 (42)	15.11 (113)	0.14	0.74 [0.49 - 1.11]
	Ins^1^	64.6 (434)	61.0 (913)	–	Ref.
	Del^2^	35.4 (238)	39 (583)	0.11	0.86 [0.71 - 1.04]
rs77830930	GG	50.5 (171)	51.9 (396)	–	Ref.
	GA	45.4 (154)	40.0 (305)	0.25	1.17 [0.90 - 1.52]
	AA	4.1 (14)	8.1 (62)	0.03*	0.53 [0.28 - 0.96]
	G	73.2 (496)	71.9 (1097)	–	Ref.
	A	26.8 (182)	28.1 (429)	0.54	0.94 [0.77 - 1.15]
rs1598856	GG	26.5 (90)	26.9 (205)	–	Ref.
	GA	47.9 (163)	50.1 (381)	0.87	0.97 [0.72 - 1.33]
	AA	25.6 (87)	23.0 (175)	0.50	1.13 [0.79 - 1.62]
	G	50.4 (343)	52.0 (791)	–	Ref.
	A	49.6 (337)	48.0 (731)	0.51	1.06 [0.89 - 1.27]
rs7340881	CC	71.8 (244)	75.1 (573)	–	Ref.
	CT	26.5 (90)	23.1 (176)	0.22	1.20 [0.89 - 1.61]
	TT	1.8 (6)	1.8 (14)	0.99	1.00 [0.38 - 2.65]
	C	85.0 (578)	86.6 (1322)	–	Ref.
	T	15.0 (102)	13.4 (204)	0.31	1.14 [0.88 - 1.48]
rs4648055	GG	58.6 (198)	51.5 (393)	–	Ref.
	GA	35.2 (119)	39.5 (301)	0.08	0.78 [0.60 - 1.03]
	AA	6.2 (21)	9.0 (69)	0.06	0.60 [0.36 - 1.02]
	G	76.2 (515)	71.2 (1087)	–	Ref.
	A	23.8 (161)	28.8 (439)	0.02*	0.77 [0.63 - 0.95]
rs4648090	GG	69.7 (237)	72.3 (550)	–	Ref.
	GA	28.2 (96)	25.4 (193)	0.33	1.15 [0.86 - 1.54]
	AA	2.1 (7)	2.4 (18)	0.82	0.90 [0.37 -2.19]
	G	83.8 (663)	85.0 (1293)	–	Ref.
	A	16.2 (110)	15.1 (229)	0.50	1.10 [0.85 - 1.40]
rs230547	CC	80.2 (272)	82.6 (629)	–	Ref.
	CT	18.6 (63)	16.1 (123)	0.32	1.18 [0.85 - 1.66]
	TT	1.2 (4)	1.3 (10)	0.90	1.18 [0.28 - 2.98]
	C	89.5 (607)	90.6 (1381)	–	Ref.
	T	10.5 (71)	9.4 (143)	0.43	1.13 [0.84 -1.53]

^1^rs28362491 insertion allele (ATTGATTG); ^2^rs28362491 deletion allele (ATTG); IgAV, IgA vasculitis; SNP, single nucleotide polymorphism; OR, odds ratio; CI, confidence interval; *The statistical significance was lost after correction for multiple testing using the Benjamini–Hochberg method for a false discovery rate (FDR) of 5%.

**Table 2:** Differences in genotype and allele frequencies of *NFKBIA* between IgAV patients and healthy controls.

SNP	Genotype, % (n)/allele, % (2n)	IgAV patients	Healthy controls	P	OR [95% CI]
rs1957106	GG	57.1 (194)	54.6 (417)	–	Ref.
	GA	37.4 (127)	38.7 (296)	0.56	0.92 [0.70 - 1.21]
	AA	5.6 (19)	6.7 (51)	0.43	0.80 [0.46 - 1.34]
	G	75.7 (515)	74.0 (1130)	–	Ref.
	A	24.3 (165)	26.1 (398)	0.38	0.91 [0.74 - 1.12]
rs1050851	GG	60.4 (206)	57.9 (442)	–	Ref.
	GA	32.3 (110)	38.0 (290)	0.14	0.81 [0.62 - 1.07]
	AA	7.3 (25)	4.1 (31)	0.05	1.73 [0.99 - 3.01]
	G	76.5 (522)	76.9 (1174)	–	Ref.
	A	23.5 (160)	23.1 (352)	0.84	1.02 [0.83 - 1.27]
rs2233415	GG	44.5 (150)	47.6 (363)	–	Ref.
	GA	46.3 (156)	42.6 (325)	0.28	1.16 [0.89 - 1.52]
	AA	9.2 (31)	9.8 (75)	1.00	1.00 [0.63 - 1.58]
	G	67.7 (456)	68.9 (1051)	–	Ref.
	A	32.3 (218)	31.1 (475)	0.57	1.06 [0.87 - 1.28]
rs2233419	GG	68.9 (235)	66.1 (505)	–	Ref.
	GA	27.0 (92)	31.3 (239)	0.19	0.82 [0.62 - 1.10]
	AA	4.1 (14)	2.6 (20)	0.25	1.50 [0.75 - 3.03]
	G	82.4 (562)	81.7 (1249)	–	Ref.
	A	17.6 (120)	18.3 (279)	0.71	0.96 [0.75 - 1.21]
rs1022714	GG	57.9 (195)	60.2 (459)	–	Ref.
	GA	35.0 (118)	35.5 (271)	0.86	1.02 [0.78 - 1.34]
	AA	7.1 (24)	4.3 (33)	0.05	1.71 [0.98 - 2.98]
	G	75.4 (508)	77.9 (1189)	–	Ref.
	A	24.6 (166)	22.1 (337)	0.19	1.15 [0.93 - 1.43]
rs696	CC	38.2 (130)	37.4 (285)	–	Ref.
	CT	46.8 (159)	48.6 (370)	0.67	1.01 [0.78 - 1.24]
	TT	15.0 (51)	14.0 (107)	0.83	1.04 [0.71 - 1.55]
	C	61.6 (419)	61.7 (940)	–	Ref.
	T	38.3 (261)	38.3 (584)	0.98	1.00 [0.83 - 1.21]
rs3138055	TT	49.1 (167)	52.7 (402)	–	Ref.
	TC	41.5 (141)	41.6 (317)	0.62	1.07 [0.82 - 1.40]
	CC	9.4 (32)	5.8 (44)	0.02*	1.75 [1.07 - 2.86]
	T	69.8 (475)	73.5 (1121)	–	Ref.
	C	30.2 (205)	26.5 (405)	0.08	1.19 [0.98 - 1.46]

IgAV, IgA vasculitis; SNP, single nucleotide polymorphism; OR, odds ratio; CI, confidence interval. *The statistical significance was lost after correction for multiple testing using the Benjamini–Hochberg method for a false discovery rate (FDR) of 5%.

Furthermore, no statistically significant differences were observed between the haplotype frequencies of *NFKB1* and *NFKBIA* in IgAV patients and healthy controls (**Table 3**).

**Table 3:** Differences in haplotype frequencies of *NFKB1* and *NFKBIA* between IgAV patients and healthy controls.

Locus	Haplotype^1^, %(2n)	IgAV patients	Healthy controls	P	OR [95% CI]
*NFKB1*	(Del^2^)GGCAGC	22.4 (153)	27.9 (424)	–	Ref.
	(Ins^3^)AACGGC	26.3 (180)	27.8 (422)	0.20	1.18 [0.91 - 1.54]
	(Ins^3^)GACGGC	12.3 (84)	10.6 (162)	0.03*	1.44 [1.03 - 2.00]
	(Ins^3^)GACGGT	9.1 (62)	8.1 (123)	0.07	1.40 [0.69 - 2.02]
	(Ins^3^)GGTGAC	7.6 (52)	8.0 (121)	0.36	1.19 [0.80 - 1.75]
	(Del^2^)GGCGAC	8.0 (55)	6.0 (91)	0.01*	1.67 [1.11 - 2.49]
*NFKBIA*	AGGGGTT	17.4 (118)	17.4 (265)	–	Ref.
	GGAGACC	19.2 (131)	16.7 (255)	0.35	1.15 [0.84 - 1.58]
	GAGAGCT	14.9 (101)	15.6 (238)	0.77	0.95 [0.68 - 1.33]
	GGAGGTT	10.4 (71)	11.1 (169)	0.75	0.94 [0.65 - 1.36]
	GGGGGCT	7.1 (48)	8.6 (131)	0.33	0.82 [0.54 - 1.24]
	GGGGGTT	6.0 (41)	5.4 (83)	0.64	1.11 [0.70 - 1.74]

The table shows *NFKB1* and *NFKBIA* haplotypes with a frequency greater than 5%. **^1^** Haplotypes are arranged in the following order, *NFKB1* (rs28362491, rs77830930, rs1598856, rs7340881, rs4648055, rs4648090, and rs230547); *NFKBIA* (rs1957106, rs1050851, rs2233415, rs2233419, rs1022714, rs696, rs3138055). ^2^rs28362491 deletion allele (ATTG); ^3^rs28362491 insertion allele (ATTGATTG); IgAV, IgA vasculitis; OR, odds ratio; CI, confidence interval. *The statistical significance was lost after correction for multiple testing using the Benjamini–Hochberg method for a false discovery rate (FDR) of 5%.

### Stratified analysis of *NFKB1* and *NFKBIA* polymorphisms according to renal manifestations in IgAV

Potential differences in genotype, allele, and haplotype frequencies of *NFKB1* and *NFKBIA* between IgAV patients stratified by disease severity were subsequently investigated. Because renal manifestations represent the most severe complication of IgAV, patients were stratified according to the presence or absence of nephritis.

Our analysis showed that IgAV patients who developed renal manifestations exhibited no statistically significant differences in the genotype and allele frequencies of *NFKB1* and *NFKBIA* compared with those without this complication (**Tables 4**, **5**). Likewise, similar results were obtained when comparing the haplotype frequencies of *NFKB1* and *NFKBIA* between IgAV patients with and without nephritis (**Table 6**).

**Table 4:** Differences in genotype and allele frequencies of *NFKB1* in IgAV patients stratified by the presence or absence of renal manifestations.

SNP	Genotype, % (n)/allele, % (2n)	Presence of renal manifestations	Absence of renal manifestations	P	OR [95% CI]
rs28362491	Ins^1^/Ins^1^	38.9 (44)	43.0 (96)	–	Ref.
	Ins^1^/Del^2^	45.1 (51)	46.2 (103)	0.76	1.08 [0.66 - 1.76]
	Del^2^/Del^2^	15.9 (18)	10.8 (24)	0.17	1.64 [0.80 - 3.34]
	Ins^1^	61.7 (139)	66.1 (295)	–	Ref.
	Del^2^	38.3 (87)	33.9 (151)	0.24	1.22 [0.88 - 1.71]
rs77830930	GG	50.0 (57)	50.7 (114)	–	Ref.
	GA	43.9 (50)	46.2 (104)	0.87	0.96 [0.60- 1.53]
	AA	6.1 (7)	3.1 (7)	0.21	2.00 [0.66 - 6.02]
	G	71.9 (164)	73.8 (332)	–	Ref.
	A	28.1 (64)	26.2 (118)	0.61	1.10 [0.77 - 1.57]
rs1598856	GG	29.0 (33)	25.2 (57)	–	Ref.
	GA	44.7 (51)	49.6 (112)	0.39	0.79 [0.46 - 1.35]
	AA	26.3 (30)	25.2 (57)	0.76	0.91 [0.49 - 1.69]
	G	51.3 (117)	50.0 (226)	–	Ref.
	A	48.7 (111)	50.0 (226)	0.75	0.95 [0.69 - 1.30]
rs7340881	CC	75.4 (86)	69.9 (158)	–	Ref.
	CT	23.7 (27)	27.9 (63)	0.37	0.79 [0.47 - 1.33]
	TT	0.9 (1)	2.2 (5)	0.35	0.38 [0.04 - 3.22]
	C	87.3 (199)	83.9 (379)	–	Ref.
	T	12.7 (29)	16.1 (73)	0.24	0.76 [0.48 - 1.20]
rs4648055	GG	53.6 (60)	61.1 (138)	–	Ref.
	GA	38.4 (43)	33.6 (76)	0.28	1.30 [0.80 - 2.11]
	AA	8.0 (9)	5.3 (12)	0.24	1.73 [0.69 - 4.33]
	G	72.8 (163)	77.9 (352)	–	Ref.
	A	27.2 (61)	22.1 (100)	0.14	1.32 [0.91 - 1.91]
rs4648090	GG	67.5 (77)	70.8 (160)	–	Ref.
	GA	29.0 (33)	27.9 (63)	0.74	1.08 [0.66 - 1.80]
	AA	3.5 (4)	1.3 (3)	0.17	2.77 [0.60 - 12.80]
	G	82.0 (187)	84.7 (383)	–	Ref.
	A	18.0 (41)	15.3 (69)	0.36	1.22 [0.80 - 1.86]
rs230547	CC	79.8 (91)	80.5 (181)	–	Ref.
	CT	17.6 (20)	19.1 (43)	0.80	0.93 [0.51 - 1.67]
	TT	2.6 (3)	0.4 (1)	0.08	5.97 [0.60 - 58.15]
	C	88.6 (202)	90.0 (405)	–	Ref.
	T	11.4 (26)	10.0 (45)	0.57	1.16 [0.69 - 1.93]

^1^rs28362491 insertion allele (ATTGATTG); ^2^rs28362491 deletion allele (ATTG); IgAV, IgA vasculitis; SNP, single nucleotide polymorphism; OR, odds ratio; CI, confidence interval.

**Table 5:** Differences in genotype and allele frequencies of *NFKBIA* in IgAV patients stratified by the presence or absence of renal manifestations.

SNP	Genotype, % (n)/allele, % (2n)	Presence of renal manifestations	Absence of renal manifestations	P	OR [95% CI]
rs1957106	GG	65.8 (75)	52.7 (119)	–	–
	GA	23.7 (27)	44.2 (100)	0.001*	0.42 [0.25 - 0.72]
	AA	10.5 (12)	3.1 (7)	0.04*	2.72 [1.01 - 7.31]
	G	77.6 (177)	74.8 (338)	–	–
	A	22.4 (51)	25.2 (114)	0.41	0.85 [0.59 - 1.25]
rs1050851	GG	59.7 (68)	60.8 (138)	–	–
	GA	29.8 (34)	33.5 (76)	0.70	0.91 [0.55 - 1.50]
	AA	10.5 (12)	5.7 (13)	0.14	1.87 [0.81 - 4.35]
	G	74.6 (170)	77.5 (352)	–	–
	A	25.4 (58)	22.3 (102)	0.39	1.18 [0.81 - 1.71]
rs2233415	GG	50.5 (57)	41.5 (93)	–	–
	GA	39.8 (45)	49.6 (111)	0.09	0.66 [0.41 - 1.07]
	AA	9.7 (11)	8.9 (20)	0.79	0.90 [0.40 - 2.01]
	G	70.4 (159)	66.3 (297)	–	–
	A	29.6 (67)	33.7 (151)	0.29	0.83 [0.59 - 1.17]
rs2233419	GG	69.3 (79)	68.7 (156)	–	–
	GA	25.4 (29)	27.8 (63)	0.72	0.91 [0.54 - 1.52]
	AA	5.3 (6)	3.5 (8)	0.48	1.48 [0.50 - 4.43]
	G	82.0 (187)	82.6 (375)	–	–
	A	18.0 (41)	17.4 (79)	0.85	1.04 [0.69 - 1.58]
rs1022714	GG	61.6 (69)	56.0 (126)	–	–
	GA	31.3 (35)	36.9 (83)	0.30	0.77 [0.47 - 1.26]
	AA	7.1 (8)	7.1 (16)	0.84	0.91 [0.37 - 2.25]
	G	77.2 (173)	74.4 (335)	–	–
	A	22.8 (51)	25.6 (115)	0.43	0.85 [0.59 - 1.25]
rs696	CC	40.4 (46)	37.2 (84)	–	–
	CT	41.2 (47)	49.6 (112)	0.29	0.77 [0.47 - 1.26]
	TT	18.4 (21)	13.2 (30)	0.47	1.28 [0.66 - 2.49]
	C	61.0 (139)	62.0 (280)	–	–
	T	39.0 (89)	38.0 (172)	0.80	1.04 [0.75 - 1.45]
rs3138055	TT	54.4 (62)	46.5 (105)	–	–
	TC	36.0 (41)	44.2 (100)	0.14	0.69 [0.43 - 1.13]
	CC	9.6 (11)	9.3 (21)	0.77	0.89 [0.40 - 1.97]
	T	72.4 (165)	68.6 (310)	–	–
	C	27.6 (63)	31.4 (142)	0.31	0.83 [0.59 - 1.19]

IgAV, IgA vasculitis; SNP, single nucleotide polymorphism; OR, odds ratio; CI, confidence interval. *The statistical significance was lost after correction for multiple testing using the Benjamini–Hochberg method for a false discovery rate (FDR) of 5%.

**Table 6:** Differences in haplotype frequencies of *NFKB1* and *NFKBIA* between IgAV patients stratified by the presence renal manifestations.

Gene	Haplotype^1^, % (2n)	Presence of renal manifestations	Absence of renal manifestations	P	OR [95% CI]
*NFKB1*	(Ins^2^)AACGGC	27.6 (63)	25.6 (116)	–	Ref
	(Del^3^)GGCAGC	26.0 (59)	20.8 (94)	0.45	1.19 [0.74 - 1.90]
	(Ins^2^)GACGGC	9.2 (21)	13.9 (63)	0.10	0.61 [0.33 - 1.13]
	(Ins^2^)GACGGT	10.5 (24)	8.4 (38)	0.62	1.16 [0.61 - 2.19]
	(Del^3^)GGCGAC	8.5 (20)	7.6 (35)	0.45	1.26 [0.66 - 2.40]
	(Ins^2^)GGTGAC	9.2 (21)	6.8 (31)	0.49	1.25 [0.62 - 2.45]
*NFKBIA*	GGAGACC	16.4 (37)	20.9 (94)	–	Ref
	AGGGGTT	15.3 (35)	19.0 (86)	0.90	1.03 [0.58 - 1.85]
	GAGAGCT	16.6 (36)	14.3 (65)	0.23	1.41 [0.78 - 2.55]
	GGAGGTT	10.1 (23)	10.5 (48)	0.54	1.22 [0.62 - 2.38]
	GGGGGCT	8.8 (20)	6.3 (29)	0.11	1.75 [0.83 - 3.66]

The table shows *NFKB1* and *NFKBIA* haplotypes with a frequency greater than 5%. **^1^** Haplotypes are arranged in the following order: *NFKB1* (rs28362491, rs77830930, rs1598856, rs7340881, rs4648055, rs4648090, and rs230547) and *NFKBIA* (rs1957106, rs1050851, rs2233415, rs2233419, rs1022714, rs696, and rs3138055). ^2^rs28362491 insertion allele (ATTGATTG); ^3^rs28362491 deletion allele (ATTG); OR, odds ratio; CI, confidence interval; IgAV, IgA vasculitis.

### Stratified analysis of *NFKB1* and *NFKBIA* polymorphisms by age at onset, articular, and GI manifestations in IgAV

Finally, we investigated whether *NFKB1* and *NFKBIA* polymorphisms were related to age at disease onset and the occurrence of articular and GI symptoms.

No statistically significant differences in the genotype, allele, or haplotype frequencies of *NFKB1* and *NFKBIA* were found between IgAV patients who developed the disease as children **(≤**20 years) and those who developed it as adults (**Supplementary Tables S3**-**S5**).

Likewise, similar genotype and allele frequencies of *NFKB1* and *NFKBIA* were observed in IgAV patients who developed articular or GI manifestations compared with those who did not (**Supplementary Tables S3**, **S4**). In addition, haplotype analysis did not reveal any further differences in haplotype frequencies between these patient groups (**Supplementary Table S5**).

## Discussion

NF-κB activity is critical for the activation and survival of B lymphocytes (20–22). *NFKB1* and *NFKBIA*, genes of the canonical NF-κB activation pathway, have been proposed as risk *loci* for many immune-mediated diseases (24, 27–31). Despite the major role that B cells play in IgAV, the involvement of these genes in this condition is still unknown. Accordingly, we evaluated, for the first time, the implication of *NFKB1* and *NFKBIA* in the pathogenesis of IgAV. For this purpose, we examined most of the variability of *NFKB1* and *NFKBIA* in a large and well-characterized series of 343 Caucasian IgAV patients.

Our results showed no significant differences in genotype, allele, or haplotype frequencies between IgAV patients and healthy individuals, suggesting that neither *NFKB1* nor *NFKBIA* is associated with susceptibility to IgAV. In this regard, there are no previous studies on the involvement of *NFKB1* and *NFKBIA* in IgAV, which emphasizes the importance of our findings. In line with our data, a study in a Spanish cohort with giant cell arteritis (35), a large-vessel vasculitis (36), found no association between the *NFKB1* polymorphism −94 ins/del ATTG (rs28362491) and disease susceptibility. Accordingly, we could hypothesize that the evaluation of polymorphisms in these genes does not appear to be useful for identifying these systemic vasculitides in Caucasian populations in clinical practice. Our results show, for the first time, that *NFKB1* and *NFKBIA* may not constitute suitable biomarkers for IgAV susceptibility.

In the context of IgAV severity, we found no association between *NFKB1* and *NFKBIA* polymorphisms and the presence of renal manifestations, suggesting that these genes may not be associated with the risk of developing nephritis in IgAV. No previous studies have examined the implication of genes in the NF-κB1 pathway in IgA-mediated renal inflammatory diseases in Caucasian populations. Consequently, a novel finding has emerged from our study, revealing that *NFKB1* and *NFKBIA* may not be useful in clinical practice for predicting renal damage in IgAV.

Regarding the implication of *NFKB1* and *NFKBIA* in other demographic and clinical manifestations of IgAV, we observed no relationship between polymorphisms in these genes and the age at disease onset or the development of articular manifestations. Concerning the latter, and in agreement with our findings, no influence of *NFKB1* and *NFKBIA* on the pathogenesis of rheumatoid arthritis—the prototypical articular inflammatory disease—has been reported in Spanish patients (37, 38). Furthermore, no involvement of *NFKB1* and *NFKBIA* polymorphisms in the development of GI complications was detected in our cohort of IgAV patients. In this context, GI symptoms of IgAV can be difficult to distinguish from those of Crohn’s disease (CD) and ulcerative colitis (UC) (39–41). Interestingly, *NFKB1* −94 ins/del ATTG (rs28362491) and *NFKBIA* rs696 have previously been linked to an increased risk of CD and UC, respectively (28, 42, 43). Therefore, and supported by our findings, polymorphisms in *NFKB1* and *NFKBIA* may serve as potential biomarkers for differentiating IgAV cases presenting only GI manifestations from CD and UC.

Our findings suggest that polymorphisms in the canonical NF-κB pathway genes *NFKB1* and *NFKBIA* are unlikely to exert a major influence on IgAV susceptibility or its clinical presentation. By using a tagging strategy, we captured most of the common variability within these loci, which strengthens the robustness of our results. While our study design and sample size provide a solid foundation, several diagnostic considerations should be acknowledged. Although IgAV was classified according to established criteria, histopathological confirmation by skin or renal biopsy was not uniformly available, as these procedures are invasive and not routinely performed in all patients. These diagnostic limitations should be considered when interpreting our results, although the large sample size and use of standardized criteria mitigate this concern. Interestingly, previous work from our group assessing other canonical NF-κB regulators (*IL33, IL1R1, VAV3*, and *CARD9*) (16, 44) also found no association with IgAV, further supporting a lack of contribution from the variants studied in this pathway to the disease. Nevertheless, the absence of genetic associations does not exclude the possibility that NF-κB signaling contributes to IgAV pathogenesis through mechanisms such as regulatory or epigenetic alterations or post-translational modifications.

Future studies integrating epigenomic, transcriptomic, and functional approaches will be necessary to fully delineate the contribution of NF-κB signaling to IgAV pathogenesis. Moreover, given the central role of NF-κB in immune regulation, the involvement of genes within the noncanonical pathway remains an important area for further exploration. Likewise, studies including non-Caucasian cohorts would be of interest to confirm and broaden the applicability of our findings across different ethnic backgrounds.

## Conclusions

In conclusion, in a large and well-characterized Caucasian cohort of IgAV patients representing the full clinical spectrum of the disease, our results do not support an association between common *NFKB1* and *NFKBIA* polymorphisms and IgAV susceptibility or severity, thereby contributing to refining the genetic landscape of this vasculitis.

## Data Availability

The original contributions presented in the study are included in the article/**Supplementary Material**, further inquiries can be directed to the corresponding author/s.
