# Supplementary material for: The role of NFKB1 and NFKBIA in immunoglobulin A vasculitis
Source: Front Immunol. 2025 Oct 24;16:1692908. doi: 10.3389/fimmu.2025.1692908 (PMC12592155; doi:10.3389/fimmu.2025.1692908)
Supplement: Supplementary file 1 [file DataSheet1.docx]

**Supplementary material**

| **Supplementary Table S1.** Context sequences of the Applied Biosystems TaqMan assays for the genetic variants studied. | | |
| --- | --- | --- |
| **ID SNP** | **Gene** | **Context sequence** |
| rs28362491 | *NFKB1* | CGTGCTGCCTGCGTTCCCCGACC[ATTG/-]ATTGGGCCCGGCAGGCGCTTCCTG |
| rs77830930 | *NFKB1* | ACAAAGAGACCTGACAGTGCATTTG[G/A]GTTAAAGAACAAAGTTTATTTCTTT |
| rs1598856 | *NFKB1* | CCTCTTTTTGTGCTGCGTTATCCAT[A/G]GCCCAGGGTTTGGGGACGCCTGCAC |
| rs7340881 | *NFKB1* | GAGAAAATTAAATAGTGCCTTTGTC[C/T]TCAGGAAACAATCCATTTAATAGAG |
| rs4648055 | *NFKB1* | GTGTGTATAGAGTCATAGGTGCAAC[A/G]TTTACCTGAGATGTTAACATAAACA |
| rs4648090 | *NFKB1* | AGAATCATGGCACTTTTGTTAATTG[A/G]TTTCTATTGAGTACTTTATTTAAAT |
| rs230547 | *NFKB1* | TTCAGTCCATTCTCTGCCCAACAGA[C/T]GGTATGATTCTTCTAAAAAATCAGT |
| rs3138055 | *NFKBIA* | AATCAACGGGATGACAGAATGACAA[C/T]GGAGAGGTCTCCAACCACAGGCCAA |
| rs696 | *NFKBIA* | CCTACCACAATAAGACGTTTTGGGC[C/T]AGGCAGTGTGCAGTGTGGATATAAG |
| rs1022714 | *NFKBIA* | AATATAATGAACTTTACAGGCTGAG[A/G]GAGTTTGCCTTTAATGCTTCTCTTT |
| rs2233419 | *NFKBIA* | CGAGGAGCCTGACTCAGTGCGTCGG[A/G]GGCAGGAAGCACCAACCTGAGCATT |
| rs2233415 | *NFKBIA* | CTGATGTGAAGTAAAAGGTGAGGGT[A/G]AATAGTGCTCAGTGGCCCTGAATTC |
| rs1050851 | *NFKBIA* | GCAGGTTGTTCTGGAAGTTGAGGAA[A/G]GCCAGGTCTCCCTTCACCTGGCGGA |
| rs1957106 | *NFKBIA* | AGTCCAGGCCGCTGTCGTGGCGGTC[A/G]TCCAGTAGCCGCTCCTTCTTCAGCC |
| Context sequences are shown as provided by Applied Biosystems for each TaqMan SNP Genotyping Assay. Orientation may vary (forward or reverse) depending on assay design. | | |

| **Supplementary Table S2.** Demographic and clinical characteristics of IgAV patients. | | |
| --- | --- | --- |
| Children (age ≤20 years old)/adults (age >20 years old), % (n) | | 71.7 (246) / 28.3 (97) |
| Females, % (n) | | 50.1 (172) |
| Age at disease onset (years, median [IQR]) | | 7 [5 - 19] |
| Duration of follow up (years, median [IQR]) | | 1 [0.2 - 1.8] |
| Palpable purpura and/or maculopapular rash, % (n) | | 100.0 (343) |
| Arthralgia and/or arthritis, % (n) | | 55.1 (189) |
| Gastrointestinal manifestations (if “a” and/or “b”), % (n) | | 51.0 (175) |
|  | (a) Bowel angina, % (n) | 48.4 (166) |
|  | (b) Gastrointestinal bleeding, % (n) | 14.6 (50) |
| Renal manifestations (if any of the following characteristics), % (n) | | 33.2 (114) |
|  | (a) Haematuria ^1^, % (n) | 31.2 (107) |
|  | (b) Proteinuria ^1^, % (n) | 28.9 (99) |
|  | (c) Nephrotic syndrome ^1^, % (n) | 4.4 (15) |
|  | (d) Renal sequelae (persistent renal involvement) ^2^, % (n) | 5.0 (17) |
| IgAV: IgA vasculitis; IQR: interquartile range. ^1^ At any time over the clinical course of the disease; ^2^ at last follow up. | | |

| **Supplementary Table S3.** Differences in the genotype and allele frequencies of *NFKB1* polymorphisms in IgAV patients stratified by age at onset and non-renal clinical features (articular and GI manifestations). | | | | | | | | | | | | | |
| --- | --- | --- | --- | --- | --- | --- | --- | --- | --- | --- | --- | --- | --- |
|  |  | **Age of onset of the disease** | | | | **Articular manifestations** | | | | **GI Manifestations** | | | |
| **SNP** | **Genotype, % (n) /allele, % (2n)** | **Children (age ≤20 years old)** | **Adults (age >20 years old)** | **P** | **OR [95% CI]** | **Yes** | **No** | **P** | **OR [95% CI]** | **Yes** | **No** | **P** | **OR [95% CI]** |
| rs28362491 | Ins^1^/Ins^1^ | 41.9 (101) | 41.1 (39) | - | Ref. | 41.3 (76) | 42.1 (64) | - | Ref. | 43.0 (74) | 40.2 (66) | - | Ref. |
|  | Ins^1^/Del^2^ | 43.2 (104) | 52.6 (50) | 0.39 | 0.80 [0.48 - 1.33] | 46.7 (86) | 44.7 (68) | 0.79 | 1.06 [0.67 - 1.69] | 44.2 (76) | 47.6 (78) | 0.55 | 0.87 [0.55 - 1.38] |
|  | Del^2^/ Del^2^ | 14.9 (36) | 6.3 (6) | 0.07 | 2.32 [0.90 - 5.99] | 12.0 (22) | 13.2 (20) | 0.83 | 0.93 [0.46 - 1.85] | 12.8 (22) | 12.2 (20) | 0.96 | 0.98 [0.49 - 1.96] |
|  | Ins^1^ | 63.5 (306) | 67.4 (128) | - | Ref. | 64.7 (238) | 64.5 (196) | - | Ref. | 65.1 (224) | 64.0 (210) | - | Ref. |
|  | Del^2^ | 36.5 (176) | 32.6 (62) | 0.34 | 1.19 [0.83 - 1.69] | 35.3 (130) | 35.5 (108) | 0.96 | 0.99 [0.72 - 1.36] | 34.9 (120) | 36.0 (118) | 0.77 | 0.95 [0.69 - 1.31] |
| rs77830930 | GG | 49.6 (121) | 52.6 (50) | - | Ref. | 47.6 (89) | 53.9 (82) | - | Ref. | 43.7 (76) | 57.6 (95) | - | Ref. |
|  | GA | 45.5 (111) | 45.3 (43) | 0.79 | 1.03 [0.66 - 1.62] | 46.5 (87) | 44.1 (67) | 0.37 | 1.20 [0.77 - 1.86] | 52.3 (91) | 38.2 (63) | 0.008* | 1.81 [1.16 - 2.82] |
|  | AA | 4.9 (12) | 2.1 (2) | 0.23 | 2.53 [0.55 - 11.7] | 5.9 (11) | 2.0 (3) | 0.06 | 3.38 [0.90 - 12.7] | 4.0 (7) | 4.2 (7) | 0.69 | 1.25 [0.42 - 3.73] |
|  | G | 72.3 (353) | 75.3 (143) | - | Ref. | 70.9 (265) | 76.0 (231) | - | Ref. | 69.8 (243) | 76.7 (253) | - | Ref. |
|  | A | 27.7 (135) | 24.7 (47) | 0.44 | 1.16 [0.98 - 1.71] | 29.1 (109) | 24.0 (73) | 0.13 | 1.30 [0.92 - 1.84] | 30.2 (105) | 23.3 (77) | 0.045* | 1.42 [1.00 - 2.00] |
| rs1598856 | GG | 28.2 (69) | 22.1 (21) | - | Ref. | 23.3 (44) | 30.5 (46) | - | Ref. | 23.4 (41) | 29.7 (49) | - | Ref. |
|  | GA | 46.1 (113) | 52.6 (50) | 0.21 | 0.69 [0.38 - 1.25] | 49.7 (94) | 45.7 (69) | 0.18 | 1.42 [0.85 - 2.40] | 50.9 (89) | 44.9 (74) | 0.17 | 1.43 [0.85 - 2.42] |
|  | AA | 25.7 (63) | 25.3 (24) | 0.52 | 0.80 [0.40 - 1.58] | 27.0 (51) | 23.8 (36) | 0.20 | 1.48 [0.81 - 2.69] | 25.7 (45) | 25.4 (42) | 0.41 | 1.28 [0.71 - 2.32] |
|  | G | 51.2 (251) | 48.4 (92) | - | Ref. | 48.2 (182) | 53.3 (161) | - | Ref. | 48.9 (171) | 52.1 (172) | - | Ref. |
|  | A | 48.8 (239) | 51.6 (98) | 0.51 | 0.89 [0.64 - 1.25] | 51.8 (196) | 46.7 (141) | 0.18 | 1.23 [0.91 - 1.67] | 51.1 (179) | 47.9 (158) | 0.39 | 1.14 [0.84 - 1.54] |
| rs7340881 | CC | 71.5 (175) | 72.6 (69) | - | Ref. | 73.5 (139) | 69.5 (105) | - | Ref. | 72.0 (126) | 71.5 (118) | - | Ref. |
|  | CT | 26.5 (65) | 26.3 (25) | 0.93 | 1.03 [0.60 - 1.76] | 25.4 (48) | 27.8 (42) | 0.55 | 0.86 [0.53 - 1.40] | 26.9 (47) | 26.1 (43) | 0.92 | 1.02 [0.63 - 1.66] |
|  | TT | 2.0 (5) | 1.1 (1) | 0.53 | 1.97 [0.22- 17.28] | 1.1 (2) | 2.7 (4) | 0.25 | 0.38 [0.07 - 2.12] | 1.1 (2) | 2.4 (4) | 0.38 | 0.47 [0.08 - 2.62] |
|  | C | 84.7 (415) | 85.8 (163) | - | Ref. | 86.2 (326) | 83.4 (252) | - | Ref. | 85.4 (299) | 84.5 (279) | - | Ref. |
|  | T | 15.3 (75) | 14.2 (27) | 0.72 | 1.09 [0.68 - 1.76] | 13.8 (52) | 16.6 (50) | 0.31 | 0.80 [0.53 - 1.23] | 14.6 (51) | 15.5 (51) | 0.75 | 0.93 [0.61 - 1.42] |
| rs4648055 | GG | 59.6 (146) | 55.9 (52) | - | Ref. | 57.7 (108) | 59.6 (90) | - | Ref. | 56.9 (99) | 60.4 (99) | - | Ref. |
|  | GA | 32.6 (80) | 41.9 (39) | 0.22 | 0.73 [0.44 - 1.20] | 38.0 (71) | 31.8 (48) | 0.37 | 1.23 [0.78 - 1.96] | 36.2 (63) | 34.1 (56) | 0.61 | 1.13 [0.71 - 1.78] |
|  | AA | 7.8 (19) | 2.2 (2) | 0.09 | 3.38 [0.75 - 15.5] | 4.3 (8) | 8.6 (13) | 0.15 | 0.51 [0.20 - 1.30] | 6.9 (12) | 5.5 (9) | 0.53 | 1.33 [0.54 - 3.32] |
|  | G | 75.9 (372) | 76.9 (143) | - | Ref. | 76.7 (287) | 75.5 (228) | - | Ref. | 75.0 (261) | 77.4 (254) | - | Ref. |
|  | A | 24.1 (118) | 23.1 (43) | 0.79 | 1.05 [0.71 - 1.57] | 23.3 (87) | 24.5 (74) | 0.71 | 0.93 [0.65 - 1.33] | 25.0 (87) | 22.6 (74) | 0.46 | 1.14 [0.80 - 1.63] |
| rs4648090 | GG | 67.1 (165) | 76.6 (72) | - | Ref. | 70.2 (132) | 69.1 (105) | - | Ref. | 71.8 (125) | 71.8 (112) | - | Ref. |
|  | GA | 30.9 (76) | 21.3 (20) | 0.08 | 1.66 [0.94 - 2.93] | 27.7 (52) | 28.9 (44) | 0.80 | 0.94 [0.58 - 1.51] | 26.5 (46) | 30.1 (50) | 0.43 | 0.82 [0.51 - 1.33] |
|  | AA | 2.0 (5) | 2.1 (2) | 0.92 | 1.09 [0.21 - 5.77] | 2.1 (4) | 2.0 (3) | 0.94 | 1.06 [0.23 - 4.86] | 1.7 (3) | 2.4 (4) | 0.61 | 0.67 [0.15 - 3.08] |
|  | G | 82.5 (406) | 87.2 (164) | - | Ref. | 84.0 (316) | 83.6 (254) | - | Ref. | 85.1 (296) | 82.5 (274) | - | Ref. |
|  | A | 17.5 (86) | 12.8 (24) | 0.14 | 1.45 [0.89 - 2.36] | 16.0 (60) | 16.4 (50) | 0.86 | 0.96 [0.64 - 1.45] | 14.9 (52) | 17.5 (58) | 0.37 | 0.83 [0.55 - 1.25] |
| rs230547 | CC | 82.4 (201) | 74.7 (71) | - | Ref. | 79.7 (149) | 80.9 (123) |  |  | 77.6 (135) | 83.0 (137) | - | Ref. |
|  | CT | 16.8 (41) | 23.2 (22) | 0.16 | 0.66 [0.37 - 1.18] | 18.7 (35) | 18.4 (28) | 0.91 | 1.03 [0.59 - 1.79] | 20.7 (36) | 16.4 (27) | 0.28 | 1.35 [0.78- 2.36] |
|  | TT | 0.8 (2) | 2.1 (2) | 0.28 | 0.35 [0.05 - 2.57] | 1.6 (3) | 0.7 (1) | 0.42 | 2.48 [0.25 - 24.3] | 1.7 (3) | 0.6 (1) | 0.31 | 3.04 [0.31 - 29.9] |
|  | C | 90.8 (443) | 86.3 (164) | - | Ref. | 89.0 (333) | 90.1 (274) | - | Ref. | 87.9 (306) | 91.2 (301) | - | Ref. |
|  | T | 9.2 (45) | 13.7 (26) | 0.09 | 0.64 [0.38 - 1.07] | 11.0 (41) | 9.9 (30) | 0.64 | 1.12 [0.68 - 1.85] | 12.1 (42) | 8.8 (29) | 0.16 | 1.42 [0.86 - 2.35] |
| ^1^rs28362491 insertion allele (ATTGATTG); ^2^rs28362491 deletion allele (ATTG); IgAV: IgA Vasculitis; GI: gastrointestinal; SNP: single nucleotide polymorphism; OR: Odds Ratio; CI: Confidence Interval. *The statistical significance was lost after correcting for multiple testing using the Benjamini-Hochberg method for a False Discovery Rate (FDR) of 5%. | | | | | | | | | | | | | |

| **Supplementary Table S4.** Differences in the genotype and allele frequencies of *NFKBIA* polymorphisms in IgAV patients stratified by age at onset and non-renal clinical features (articular and GI manifestations). | | | | | | | | | | | | | |
| --- | --- | --- | --- | --- | --- | --- | --- | --- | --- | --- | --- | --- | --- |
|  |  | **Age of onset of the disease** | | | | **Articular manifestations** | | | | **GI Manifestations** | | | |
| **SNP** | **Genotype, % (n) /allele, % (2n)** | **Children (age ≤20 years old)** | **Adults (age >20 years old)** | **P** | **OR [95% CI]** | **Yes** | **No** | **P** | **OR [95% CI]** | **Yes** | **No** | **P** | **OR [95% CI]** |
| rs1957106 | GG | 57.2 (139) | 56.7 (55) | - | - | 52.9 (99) | 62.1 (95) | - | Ref. | 57.5(100) | 56.6 (94) | - | - |
|  | GA | 37.0 (90) | 38.1 (37) | 0.88 | 0.96 [0.59 - 1.58] | 40.1 (75) | 34.0 (52) | 0.16 | 1.38 [0.88 - 2.18] | 36.8 (64) | 38.0 (63) | 0.84 | 0.95 [0.61 - 1.50] |
|  | AA | 5.8 (14) | 5.2 (5) | 0.85 | 1.11 [0.38 - 3.23] | 7.0 (13) | 3.9 (6) | 0.15 | 2.08 [0.75 - 5.74] | 5.7 (10) | 5.4 (9) | 0.93 | 1.04 [0.41 - 2.69] |
|  | G | 75.7 (368) | 75.8 (147) | - | - | 73.0 (273) | 79.1 (242) | - | Ref. | 75.9 (264) | 75.6 (251) | - | - |
|  | A | 24.3 (118) | 24.2 (47) | 0.99 | 1.00 [0.68 - 1.48] | 27.0 (101) | 20.9 (64) | 0.065 | 1.40 [0.98 - 2.00] | 24.1 (84) | 24.4 (81) | 0.94 | 0.99 [0.69 - 1.40] |
| rs1050851 | GG | 55.3 (136) | 73.7 (70) | - | - | 60.8 (115) | 59.9 (91) | - | Ref. | 61.1 (107) | 59.6 (99) | - | - |
|  | GA | 37.0 (91) | 20.0 (19) | 0.002* | 2.47 [1.38 - 4.41] | 32.3 (61) | 32.2 (49) | 0.95 | 0.98 [0.62 - 1.57] | 33.2 (58) | 32.3 (52) | 0.89 | 1.03 [0.65 - 1.64] |
|  | AA | 7.7 (19) | 6.3 (6) | 0.32 | 1.63 [0.62 - 4.28] | 6.9 (13) | 7.9 (12) | 0.72 | 0.86 [0.37 - 1.97] | 5.7 (10) | 9.1 (15) | 0.26 | 0.62 [0.26 - 1.44] |
|  | G | 73.8 (363) | 83.7 (159) | - | - | 77.0 (291) | 76.0 (231) | - | Ref. | 77.7 (272) | 75.3 (250) | - | - |
|  | A | 26.2 (129) | 16.3 (38) | 0.006* | 1.82 [1.78 - 2.82] | 23.0 (87) | 24.0 (73) | 0.76 | 0.94 [0.66 - 1.35] | 22.3 (78) | 24.7 (82) | 0.46 | 0.87 [0.61 - 1.25] |
| rs2233415 | GG | 46.3 (112) | 40.0 (38) | - | - | 44.0 (81) | 45.1 (69) | - | Ref. | 40.1 (69) | 49.1 (81) | - | - |
|  | GA | 45.4 (110) | 48.4 (46) | 0.42 | 0.81 [0.49 - 1.34] | 47.8 (88) | 44.4 (68) | 0.67 | 1.10 [0.70 - 1.73] | 48.3 (83) | 44.2 (73) | 0.21 | 1.33 [0.85 - 2.10] |
|  | AA | 8.3 (20) | 11.6 (11) | 0.25 | 0.62 [0.27 - 1.41] | 8.2 (15) | 10.5 (16) | 0.57 | 0.80 [0.37 - 1.74] | 11.6 (20) | 6.7 (11) | 0.06 | 2.13 [0.94 - 4.81] |
|  | G | 69.0 (334) | 64.2 (122) | - | - | 67.9 (250) | 67.3 (206) | - | Ref. | 64.2 (221) | 71.2 (235) | - | - |
|  | A | 31.0 (150) | 35.8 (68) | 0.23 | 0.81 [0.56 - 1.15] | 32.1 (118) | 32.7 (100) | 0.87 | 0.97 [0.70 - 1.34] | 35.8 (123) | 28.8 (95) | 0.053 | 1.38 [0.99 - 1.91] |
| rs2233419 | GG | 66.7 (164) | 74.7 (71) | - | - | 66.1 (125) | 72.4 (110) | - | Ref. | 67.4 (118) | 70.5 (117) | - | - |
|  | GA | 29.7 (73) | 20.0 (19) | 0.08 | 1.66 [0.93 - 2.97] | 30.7 (58) | 22.4 (34) | 0.11 | 1.50 [0.91 - 2.47] | 29.2 (51) | 24.7 (41) | 0.40 | 1.23 [0.76 - 2.00] |
|  | AA | 3.6 (9) | 5.3 (5) | 0.66 | 0.78 [0.25 - 2.41] | 3.2 (6) | 5.3 (8) | 0.45 | 0.66 [0.22 - 1.97] | 3.4 (6) | 4.8 (8) | 0.59 | 0.74 [0.25 - 2.22] |
|  | G | 81.5 (401) | 84.7 (161) | - | - | 81.5 (308) | 83.5 (254) | - | Ref. | 82.0 (287) | 82.8 (275) | - | - |
|  | A | 18.5 (91) | 15.3 (29) | 0.32 | 1.26 [0.80 - 1.99] | 18.5 (70) | 15.5 (50) | 0.48 | 1.15 [0.77 - 1.72] | 18.0 (63) | 17.2 (57) | 0.78 | 1.06 [0.71- 1.57] |
| rs1022714 | GG | 59.8 (146) | 52.7 (49) | - | - | 60.3 (111) | 54.9 (84) | - | Ref. | 54.1 (93) | 61.8 (102) | - | - |
|  | GA | 34.0 (83) | 37.6 (35) | 0.38 | 0.80 [0.48 - 1.33] | 33.2 (61) | 37.3 (57) | 0.37 | 0.81 [0.51 - 1.28] | 37.2 (64) | 32.7 (54) | 0.26 | 1.18 [0.75 - 1.87] |
|  | AA | 6.2 (15) | 9.7 (9) | 0.20 | 0.56 [0.23 - 1.37] | 6.5 (12) | 7.8 (12) | 0.52 | 0.76 [0.33 - 1.77] | 8.7 (15) | 5.5 (9) | 0.17 | 1.97 [0.82 - 4.74] |
|  | G | 76.8 (375) | 71.5 (133) | - | - | 76.9 (283) | 73.5 (225) | - | Ref. | 72.7 (250) | 78.2 (258) | - | - |
|  | A | 23.2 (113) | 28.5 (53) | 0.15 | 0.76 [0.52 - 1.11] | 23.1 (85) | 26.5 (81) | 0.31 | 0.83 [0.59 - 1.19] | 27.3 (94) | 21.8 (72) | 0.1 | 1.35 [0.95 - 1.92] |
| rs696 | CC | 39.0 (96) | 36.2 (34) | - | - | 37.8 (71) | 38.8 (59) | - | Ref. | 39.1 (68) | 37.3 (62) | - | - |
|  | CT | 46.4 (114) | 47.9 (45) | 0.68 | 0.90 [0.53 - 1.51] | 47.3 (89) | 46.1 (70) | 0.82 | 1.06 [0.66 - 1.69] | 47.7 (83) | 45.8 (76) | 0.98 | 1.00 [0.63 - 1.58] |
|  | TT | 14.6 (36) | 15.9 (15) | 0.66 | 0.85 [0.41 - 1.75] | 14.9 (28) | 15.1 (23) | 0.92 | 1.01 [0.53 - 1.94] | 13.2 (23) | 16.9 (28) | 0.38 | 0.75 [0.39 - 1.44] |
|  | C | 62.2 (306) | 60.1 (113) | - | - | 61.4 (231) | 61.8 (188) | - | Ref. | 62.9 (219) | 60.2 (200) | - | - |
|  | T | 37.8 (186) | 39.9 (75) | 0.62 | 0.92 [0.65 - 1.29] | 38.6 (145) | 38.2 (116) | 0.91 | 1.01 [0.75 - 1.39] | 37.1 (129) | 39.8 (132) | 0.47 | 0.89 [0.65 - 1.22] |
| rs3138055 | TT | 51.6 (126) | 42.7 (41) | - | Ref. | 51.3 (96) | 46.4 (71) | - | Ref. | 47.1 (82) | 51.2 (85) | - | - |
|  | TC | 40.2 (98) | 44.8 (43) | 0.24 | 0.74 [0.45 - 1.23] | 39.6 (74) | 43.8 (67) | 0.38 | 0.82 [0.52 - 1.28] | 42.0 (73) | 41.0 (68) | 0.64 | 1.11 [0.71 - 1.74] |
|  | CC | 8.2 (20) | 12.5 (12) | 0.13 | 0.54 [0.24 - 1.21] | 9.1 (17) | 9.8 (15) | 0.65 | 0.84 [0.39 - 1.79] | 10.9 (19) | 7.8 (13) | 0.29 | 1.51 [0.70 - 3.28] |
|  | T | 71.7 (350) | 65.1 (125) | - | Ref. | 71.1 (266) | 68.3 (209) | - | Ref. | 68.1 (237) | 71.7 (238) | - | - |
|  | C | 28.3 (138) | 34.9 (67) | 0.09 | 0.74 [0.51 - 1.05] | 28.9 (108) | 31.7 (97) | 0.43 | 0.87 [0.63 - 1.22] | 31.9 (111) | 28.3 (94) | 0.31 | 1.19 [0.85 - 1.65] |
| IgAV: IgA Vasculitis; GI: gastrointestinal; SNP: single nucleotide polymorphism; OR: Odds Ratio; CI: Confidence Interval; *The statistical significance was lost after correcting for multiple testing using the Benjamini-Hochberg method for a False Discovery Rate (FDR) of 5%. | | | | | | | | | | | | | |

| **Supplementary Table S5.** Differences in the haplotype frequencies of *NFKB1* and *NFKBIA* between IgAV patients stratified by age at onset and non-renal clinical features (articular and GI manifestations). | | | | | | | | | | | | | |
| --- | --- | --- | --- | --- | --- | --- | --- | --- | --- | --- | --- | --- | --- |
|  |  | **Age of onset of the disease** | | | | **Articular manifestations** | | | | **GI Manifestations** | | | |
|  | **Haplotype^1^, % (2n)** | **Children (age ≤20 years old)** | **Adults (age >20 years old)** | **P** | **OR [95% CI]** | **Yes** | **No** | **P** | **OR [95% CI]** | **Yes** | **No** | **P** | **OR [95% CI]** |
| ***NFKB1*** | (Ins^2^)AACGGC | 27.1 (134) | 24.2 (46) | - | Ref | 28.4 (107) | 23.7 (72) | - | Ref | 29.4 (103) | 23.2 (77) | - | Ref |
|  | (Del^3^)GGCAGC | 23.5 (116) | 20.1 (38) | 0.85 | 1.05 [0.62 - 1.78] | 23.4 (89) | 21.6 (66) | 0.66 | 0.91 [0.57 - 1.44] | 23.8 (83) | 21.2 (70) | 0.59 | 0.89 [0.56 - 1.40] |
|  | (Ins^2^)GACGGC | 12.4 (61) | 11.7 (22) | 0.87 | 0.95 [0.51 - 1.81] | 12.8 (48) | 12.1 (37) | 0.61 | 0.87 [0.50 - 1.53] | 9.3 (33) | 15.7 (52) | 0.01* | 0.47 [0.27 - 0.83] |
|  | (Ins^2^)GACGGT | 7.8 (38) | 12.6 (24) | 0.05* | 0.54 [0.28 - 1.06] | 9.4 (35) | 8.8 (27) | 0.65 | 0.87 [0.47 - 1.64] | 10.6 (37) | 7.5 (25) | 0.74 | 1.11 [0.59 - 2.09] |
|  | (Del^3^)GGCGAC | 8.8 (43) | 5.2 (10) | 0.32 | 1.48 [0.66 - 3.56] | 7.3 (28) | 8.3 (25) | 0.37 | 0.75 [0.39 - 1.47] | 6.2 (22) | 9.6 (32) | 0.03* | 0.51 [0.26 - 0.99] |
|  | (Ins^2^)GGTGAC | 7.9 (39) | 6.6 (13) | 0.94 | 1.03 [0.48 - 2.29] | 7.8 (30) | 7.7 (24) | 0.58 | 0.84 [0.44 - 1.64] | 8.2 (29) | 7.0 (23) | 0.85 | 0.94 [0.48 - 1.85] |
| ***NFKBIA*** | GGAGACC | 18.1 (84) | 22.4 (43) | - | Ref | 19.5 (73) | 18.9 (58) | - | Ref | 21.7 (75) | 16.8 (56) | - | Ref |
|  | AGGGGTT | 18.3 (89) | 15.0 (29) | 0.11 | 1.57 [0.87 - 2.86] | 19.5 (73) | 14.9 (46) | 0.37 | 1.26 [0.74 - 2.16] | 17.3 (60) | 17.9 (59) | 0.28 | 0.76 [0.45 - 1.29] |
|  | GAGAGCT | 16.5 (80) | 10.4 (20) | 0.02* | 2.05 [1.07 - 4.00] | 15.8 (59) | 13.6 (42) | 0.68 | 1.12 [0.64 - 1.95] | 15.3 (53) | 14.4 (48) | 0.47 | 0.82 [0.47 - 1.14] |
|  | GGAGGTT | 9.8 (48) | 12.2 (23) | 0.834 | 1.07 [0.55 - 2.09] | 10.1 (38) | 11.2 (34) | 0.39 | 1.31 [0.67 - 2.58] | 11.0 (38) | 9.9 (33) | 0.61 | 0.86 [0.46 - 1.60] |
|  | GGGGGCT | 8.0 (32) | 5.3 (10) | 0.22 | 1.64 [0.70 - 4.09] | 6.6 (25) | 7.6 (23) | 0.66 | 0.86 [0.42 - 1.77] | 6.1 (21) | 7.8 (26) | 0.14 | 0.60 [0.29 - 1.24] |
| The table shows the *NFKB1* and *NFKBIA* haplotypes with a frequency greater than 5%. **^1^**Haplotypes are arranged in the following order: *NFKB1* (rs28362491, rs77830930, rs1598856, rs7340881, rs4648055, rs4648090, and rs230547) and *NFKBIA* (rs1957106, rs1050851, rs2233415, rs2233419, rs1022714, rs696, and rs3138055). ^2^rs28362491 insertion allele (ATTGATTG); ^3^rs28362491 deletion allele (ATTG); OR: Odds Ratio; CI: Confidence Interval; IgAV: IgA Vasculitis; GI: gastrointestinal; *The statistical significance was lost after correcting for multiple testing using the Benjamini-Hochberg method for a False Discovery Rate (FDR) of 5%. | | | | | | | | | | | | | |
